# Heterogeneous catalase-like activity of gold(i)–cobalt(iii) metallosupramolecular ionic crystals†
†Electronic supplementary information (ESI) available: Molecular structures (Fig. S1 and S2), time profiles of O_2_ evolution and TOF (Fig. S3–S5 and S9–S12), solubility of [**1**]X_*n*_ (Tables S1 and S2), XPS (Fig. S6), PXRD (Fig. S7), NMR (Fig. S8), catalase-like activity (Table S3), microscopic photos (Fig. S13), energy profiles (Fig. S14 and S15). See DOI: 10.1039/c6sc04993a



**DOI:** 10.1039/c6sc04993a

**Published:** 2017-01-16

**Authors:** Mihoko Yamada, Nobuto Yoshinari, Naoto Kuwamura, Toru Saito, Satoshi Okada, Sai Prakash Maddala, Koji Harano, Eiichi Nakamura, Kohei Yamagami, Keisuke Yamanaka, Akira Sekiyama, Tomoyoshi Suenobu, Yusuke Yamada, Takumi Konno

**Affiliations:** a Department of Chemistry , Graduate School of Science , Osaka University , Toyonaka , Osaka 560-0043 , Japan . Email: konno@chem.sci.osaka-u.ac.jp; b Department of Biomedical Information Sciences , Graduate School of Information Sciences , Hiroshima City University , Asa-Minami-ku , Hiroshima 731-3194 , Japan; c Department of Chemistry , Graduate School of Science , The University of Tokyo , Bunkyo-ku , Tokyo 113-0033 , Japan; d Division of Materials Physics , Graduate School of Engineering Science , Osaka University , Toyonaka , Osaka 560-8531 , Japan; e Synchrotron Radiation Center , Ritsumeikan University , Kusatsu , Shiga 525-8577 , Japan; f Department of Material and Life Science , Graduate School of Engineering , Osaka University , ALCA and SENTAN , Japan Science and Technology (JST) , Suita , Osaka 565-0871 , Japan; g Department of Applied Chemistry & Bioengineering , Graduate School of Engineering , Osaka City University , Sumiyoshi-ku , Osaka 558-8585 , Japan

## Abstract

Unique heterogeneous catalase-like activity was observed for metallosupramolecular ionic crystals [Au^I^_4_Co^III^_2_(dppe)_2_(d-pen)_4_]X_*n*_ ([**1**]X_*n*_; dppe = 1,2-bis(diphenylphosphino)ethane; d-pen = d-penicillaminate; X_*n*_ = (Cl^–^)_2_, (ClO_4_^–^)_2_, (NO_3_^–^)_2_ or SO_4_^2–^) consisting of Au^I^_4_Co^III^_2_ complex cations, [**1**]^2+^, and inorganic anions, X^–^ or X^2–^. Treatment of the ionic crystals with an aqueous H_2_O_2_ solution led to considerable O_2_ evolution with a high turnover frequency of 1.4 × 10^5^ h^–1^ for the heterogeneous cobalt complexes, which was dependent on their size and shape as well as the arrangement of cationic and anionic species. These dependencies were rationalized by the presence of cobalt(ii) centers on the crystal surface and their efficient exposure on the (111) plane rather than the (100) plane based on morphological and theoretical studies.

## Abstract 2

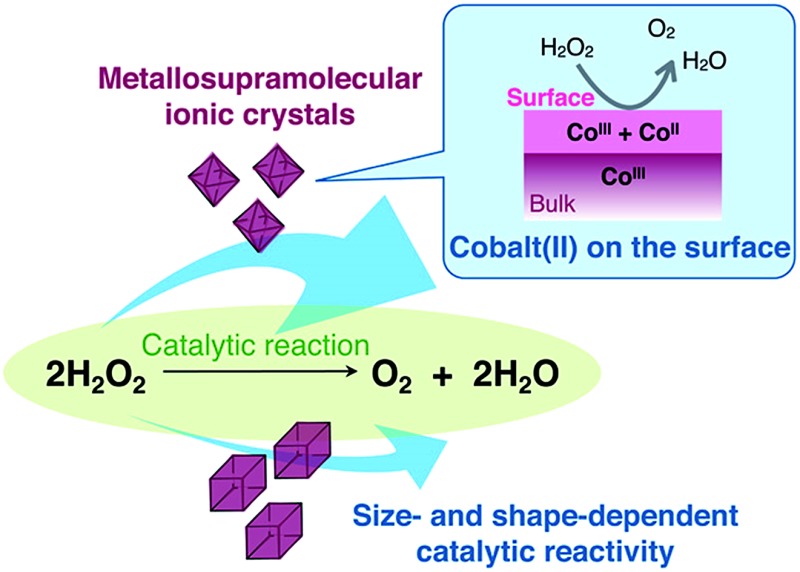
The heterogeneous catalase-like activity of ionic crystals consisting of Au^I^_4_Co^III^_2_ complex cations is studied along with their surface morphologies and oxidation states.

## Introduction

Heterogeneous catalysts have been developed for industrial applications because of their facile handling, high durability and reusability.^1^ Various approaches, such as increasing the surface area by decreasing the particle size,^2^ attachment to mesoporous carriers,^3^ introduction of heterogeneous elements, enlargement of dispersion^4^ and modification of the crystal surface, have been used to control and improve their catalytic activities.^5^ In particular, the catalytic activities of metals or metal oxides with high durability have been improved by controlling the size and shape of the crystals.^6^ Compared to metal and metal oxide catalysts, catalysts consisting of coordination compounds (metal complexes) have certain advantages with respect to the precise modulation of their structures and properties through modification of the ligands employed.^7^ However, the relationship between the size and shape of the crystals and the catalytic activities of coordination compounds has rarely been investigated.

One well-known catalytic reaction is the disproportionation of hydrogen peroxide into water and oxygen, which is similar to the catalase reaction in living organisms (eqn (1)).^8^

1 2H_2_O_2_ → O_2_ + 2H_2_O

**Fig. 1:**
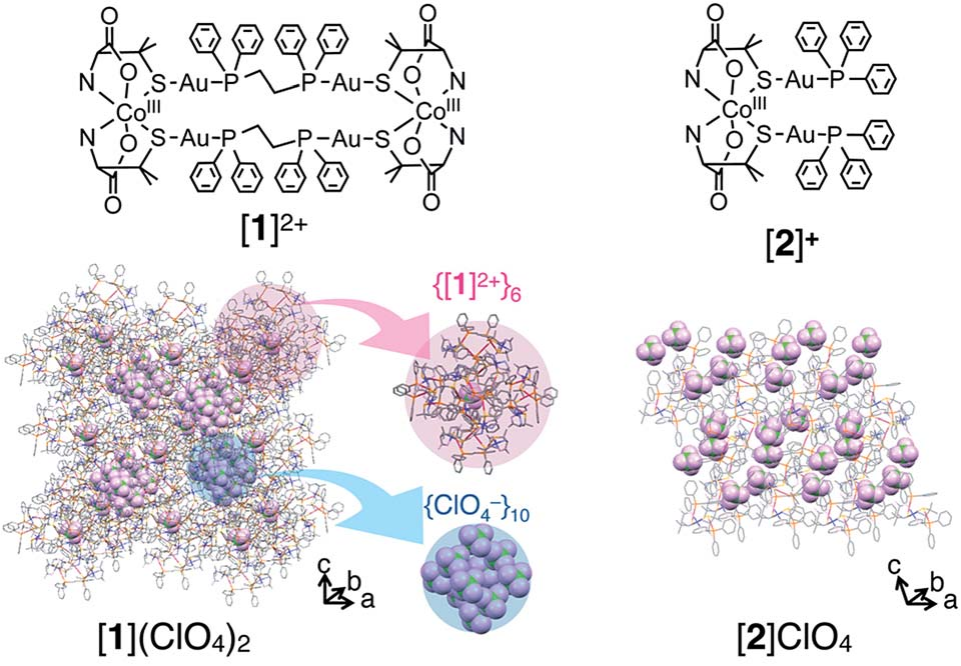
Structures of the Au^I^_4_Co^III^_2_ complex [Au^I^_4_Co^III^_2_(dppe)_2_(d-pen)_4_]^2+^ ([**1**]^2+^) and Au^I^_2_Co^III^ complex [Co^III^{Au^I^(PPh_3_)(d-pen)}_2_]^+^ ([**2**]^+^) and crystal packing structures of [**1**](ClO_4_)_2_ and [**2**]ClO_4_.^11,15^ An anion is accommodated at the center of {[**1**]^2+^}_6_. H_2_O molecules are omitted for clarity.

This catalase-like activity has been observed for many coordination compounds under homogeneous and/or heterogeneous conditions. However, only a few cobalt(ii) complexes have been reported to show catalase-like activity under heterogeneous conditions.^9^ Furthermore, no reports exist on the catalase-like activity of cobalt(iii) complexes except one report of low activity under homogeneous conditions.^10^ Herein, we report that metallosupramolecular ionic crystals containing cobalt(iii) centers, [Au^I^_4_Co^III^_2_(dppe)_2_(d-pen)_4_]X_*n*_ ([**1**]X_*n*_, dppe = 1,2-bis(diphenylphosphino)ethane, d-pen = d-penicillaminate, X_*n*_ = (Cl^–^)_2_, (ClO_4_^–^)_2_ (NO_3_^–^)_2_ or SO_4_^2–^, Fig. 1), which were recently synthesized and structurally characterized,^11^ exhibit high catalase-like activity under heterogeneous conditions. This class of ionic crystals, which we refer to as ‘non-coulombic ionic solids (NCIS)’, consists of Au^I^_4_Co^III^_2_ hexanuclear complex cations, [**1**]^2+^, and inorganic anions, X^–^ or X^2–^, and adopts an unusual non-alternate arrangement of cationic and anionic species governed by non-coulombic interactions; complex cations [**1**]^2+^ are self-assembled into octahedron-shaped cationic supramolecules, {[**1**]^2+^}_6_, and anions X^–^ or X^2–^ are aggregated into adamantane- or octahedron-shaped anionic clusters, {X^–^}_10_ or {X^2–^}_6_ (Fig. 1, S1 and S2 in the ESI†).^11^ Mechanistic insight into the appearance of catalase-like activity for crystals of [**1**]X_*n*_, together with its size and shape dependency, is also reported.

## Results and discussion

### Catalase-like activity of ionic crystals of [**1**]X_*n*_

**Fig. 2:**
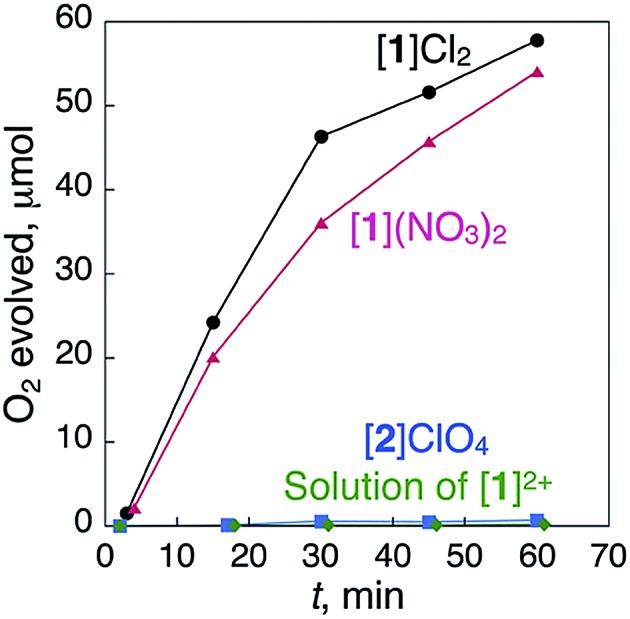
Time-dependent profiles of the evolution of O_2_ during treatment with a catalytic amount of [**1**]Cl_2_ (5.0 mg), [**1**](NO_3_)_2_ (4.9 mg), [**2**]ClO_4_ (5.0 mg) and a homogeneous saturated solution of [**1**]Cl_2_ in H_2_O (0.83 mL) with 5% aqueous H_2_O_2_ (1.00 mL total) at 298 K.

When ionic crystals of the octahedron-shaped [**1**]Cl_2_ or [**1**](NO_3_)_2_ were soaked in a 5% aqueous solution of H_2_O_2_ at room temperature without stirring, the generation of bubbles was observed within a few minutes. Based on the gas chromatography (GC) results, we confirmed that this phenomenon is due to the evolution of O_2_ gas caused by a heterogeneous catalase-like reaction (Fig. 2).^12,13^ Similar treatment of aqueous H_2_O_2_ with ionic crystals of the Au^I^_4_Cr^III^_2_ hexanuclear complexes,^11^ [Au_4_Cr_2_(dppe)_2_(d-pen)_4_]Cl_2_, which are isomorphous with [**1**]Cl_2_, showed no significant evolution of O_2_ gas (Fig. S4†). The same result was obtained when the digold(i) complex, [Au_2_(dppe)(d-Hpen)_2_],^11^ which is a precursor to the preparation of [**1**]Cl_2_ and [**1**](NO_3_)_2_, was treated with aqueous H_2_O_2_ (Fig. S5†). Thus, the cobalt centers in [**1**]Cl_2_ or [**1**](NO_3_)_2_, rather than gold centers, are the active sites of this catalytic reaction. One may suspect that Co^0^ nanoparticles that act as catalysts are produced over the course of the reaction.^14^ However, no peaks attributed to Co^0^ were detected near 778 eV in the X-ray photoelectron spectroscopy (XPS) spectra of the crystal [**1**]Cl_2_ after treatment with H_2_O_2_ (Fig. S6†),^15^ which indicates the absence of Co^0^ species.

Powder X-ray diffraction (PXRD) measurements revealed that the crystallinity of [**1**]Cl_2_ and [**1**](NO_3_)_2_ is retained after treatment with H_2_O_2_ (Fig. S7†). The durability of [**1**]Cl_2_ was revealed by the ^1^H and ^31^P NMR and XPS measurements of the samples after the reaction (Fig. S6 and S8†). Furthermore, the second reaction of the [**1**]Cl_2_ crystals, which were collected after reaction for 60 min, had an activity comparable to that in the first run, thus indicating reusability (Fig. S9†).

The turnover number (TON) and frequency (TOF) of the [**1**]Cl_2_/[**1**](NO_3_)_2_ crystals at 15 min were estimated as 3.2 × 10^4^/3.4 × 10^4^ and 1.2 × 10^5^/1.4 × 10^5^ h^–1^, respectively, assuming that only the cobalt centers located on the crystal surface are involved in the reaction.^16^ Notably, the TOF values for [**1**]Cl_2_ and [**1**](NO_3_)_2_ were much higher than that for cobalt complexes with the highest heterogeneous catalase-like activity at room temperature, as has been reported previously (5.5 × 10^3^ h^–1^).^9b,17^

To determine the effect of the arrangement of cationic and anionic species in the crystal on the appearance of the catalase-like activity, ionic crystals of [Co^III^{Au^I^(PPh_3_)(d-pen)}_2_]ClO_4_ (ref. 18) ([**2**]ClO_4_, Fig. 1), which has the same coordination environment around the Co^III^ center as that in [**1**]^2+^ but adopts a typical alternating arrangement of cations and anions in the crystal, were treated with aqueous H_2_O_2_ under the same conditions (Fig. 2). Remarkably, no evolution of O_2_ gas was observed with this treatment, which was also the case during treatment of a saturated solution of [**1**]Cl_2_ in H_2_O with aqueous H_2_O_2_.^12^ Thus, the arrangement of cationic and anionic species plays a key role in the appearance of the heterogeneous catalase-like activity of the [**1**]Cl_2_ and [**1**](NO_3_)_2_ crystals.

Since the activity of heterogeneous catalysts often depends on the surface area, we examined the catalase-like activity of [**1**]Cl_2_ and [**1**](NO_3_)_2_ using crystals of different sizes (Fig. S10 and S11†).^19^ The use of small crystals (0.4 × 0.4 × 0.4 mm for [**1**]Cl_2_ and 0.05 × 0.05 × 0.05 mm for [**1**](NO_3_)_2_) led to greater evolution of O_2_ gas compared to the use of large crystals (1.0 × 1.0 × 1.0 mm for [**1**]Cl_2_ and [**1**](NO_3_)_2_), which is expected from the larger surface area per unit mass. The TOFs at 15 min, which were calculated by considering the number of Co atoms on the crystal surface, were equivalent for the small and large crystals. Furthermore, a proportional relationship between the catalytic activities and the amounts of crystal used was observed, which is consistent with the surface-area dependency of the catalytic activities (Fig. S12†).

**Fig. 3:**
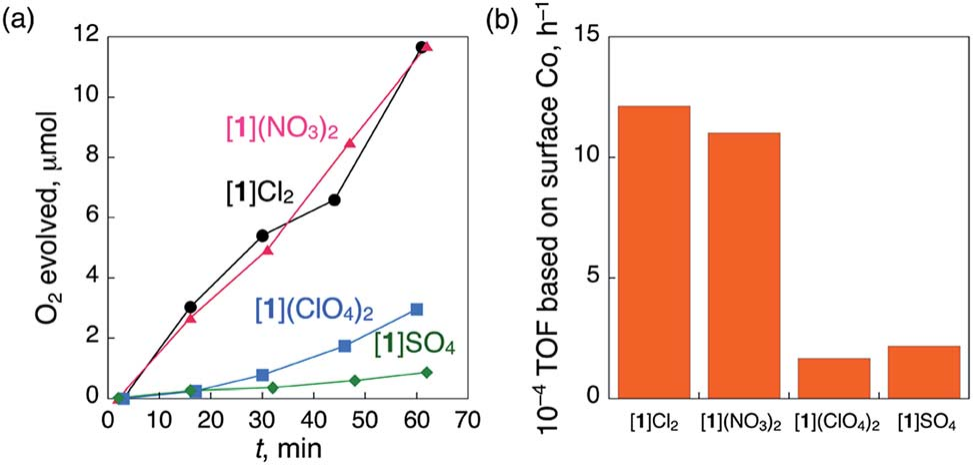
(a) Time-dependent profiles of the evolution of O_2_ during treatment with a catalytic amount of 1.0 × 1.0 × 1.0 mm-sized crystals of [**1**]Cl_2_ (5.2 mg), [**1**](NO_3_)_2_ (5.1 mg), [**1**](ClO_4_)_2_ (4.8 mg) or [**1**]SO_4_ (5.3 mg) with 5% aqueous H_2_O_2_ (1.00 mL) at 298 K and (b) TOF calculated based on the number of surface Co atoms at 15 min.

We observed that the crystal shapes of [**1**](ClO_4_)_2_ and [**1**]SO_4_ are cubic, unlike the octahedral shape of the [**1**]Cl_2_ and [**1**](NO_3_)_2_ crystals. To determine the difference in the catalase-like activities between the octahedral and cubic crystals, the same size of crystals (*ca.* 1.0 × 1.0 × 1.0 mm) of these compounds were treated with aqueous H_2_O_2_ under the same conditions, eliminating the size-dependent effect (Fig. S13†). As expected, all the crystals showed O_2_ evolution, which was confirmed by GC measurements (Fig. 3a). The TOFs at 15 min for the octahedral crystals [**1**]Cl_2_ and [**1**](NO_3_)_2_ were more than 5 times higher than those for the cubic crystals [**1**](ClO_4_)_2_ and [**1**]SO_4_ (Fig. 3b). No significant difference in the TOF values was observed between [**1**]Cl_2_ and [**1**](NO_3_)_2_ or between [**1**](ClO_4_)_2_ and [**1**]SO_4_. This result clearly indicates that the heterogeneous catalytic activity of [**1**]X_*n*_ is highly dependent on the crystal shape, rather than the type of anionic species in the crystal.

### Crystal surface morphology and oxidation state of cobalt centers of [**1**]X_*n*_

**Fig. 4:**
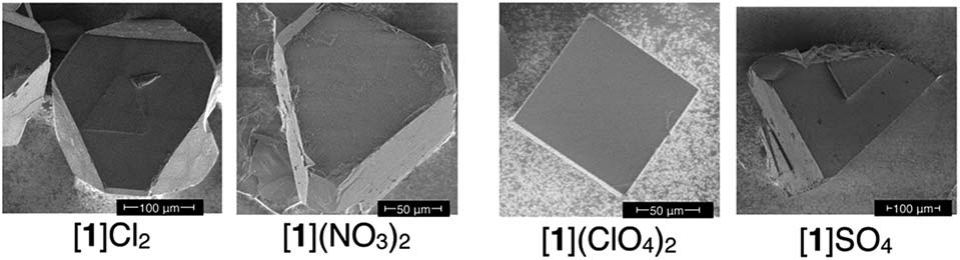
SEM images of crystals [**1**]Cl_2_, [**1**](NO_3_)_2_, [**1**](ClO_4_)_2_, and [**1**]SO_4_.

**Fig. 5:**
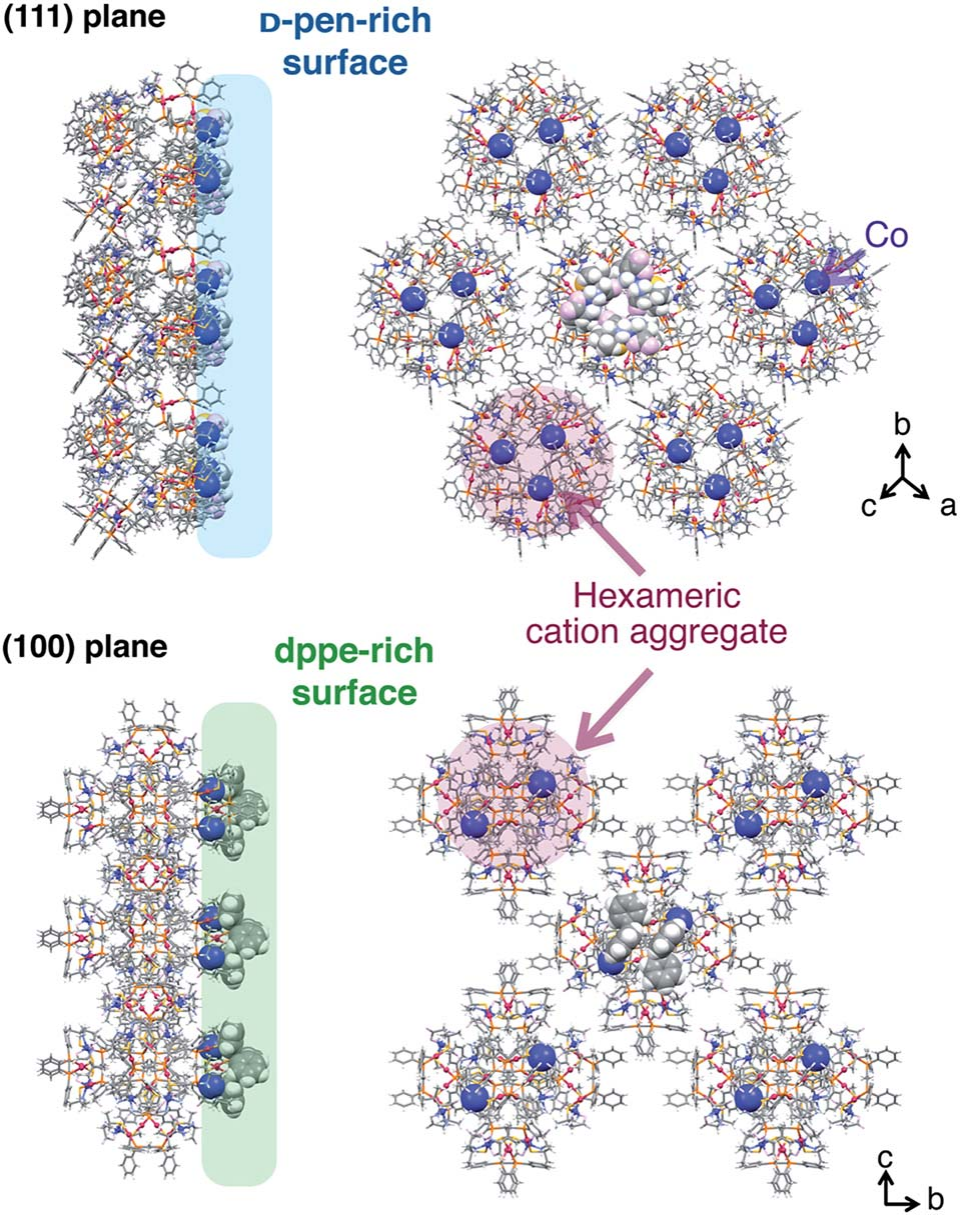
Side and top views of the arrangement of cationic supramolecular octahedrons {[**1**]^2+^}_6_ on the crystal surface of the (100) plane and the (111) plane.^11^

The crystal-shape dependency of the catalase-like activity of [**1**]X_*n*_ can be discussed from the viewpoint of the surface morphology. Scanning electron microscopy (SEM) images of the crystal surfaces revealed that the crystal growth of the (111) plane is dominant for [**1**]Cl_2_ and [**1**](NO_3_)_2_, whereas growth of the (100) plane is dominant for [**1**](ClO_4_)_2_ and [**1**]SO_4_, as expected based on their macroscopic crystal shapes (Fig. 4).^6^ Thus, it is reasonable to assume that the catalytic activity on the (111) plane is higher than that on the (100) plane, considering the higher activity of the [**1**]Cl_2_ and [**1**](NO_3_)_2_ crystals. This tendency contrasts with the catalytic activities of platinum nanoparticles, for which the (100) plane has higher activity than the (111) plane; this phenomenon has been ascribed to a looser alignment of platinum atoms on the (100) plane.^6^ The surface structure of each crystal plane of [**1**]X_*n*_, determined by single-crystal X-ray analysis, is shown in Fig. 5.^11^ The (111) plane is a d-pen-rich surface, and the cobalt centers are fully exposed by the easy dissociation of the carboxyl group of d-pen, as discussed below. On the other hand, the (100) plane is a dppe-rich surface, and the cobalt centers are much less exposed. Thus, the (111) plane is advantageous due to the exposure of the cobalt centers on the crystal surface, which leads to high catalytic activity.

**Fig. 6:**
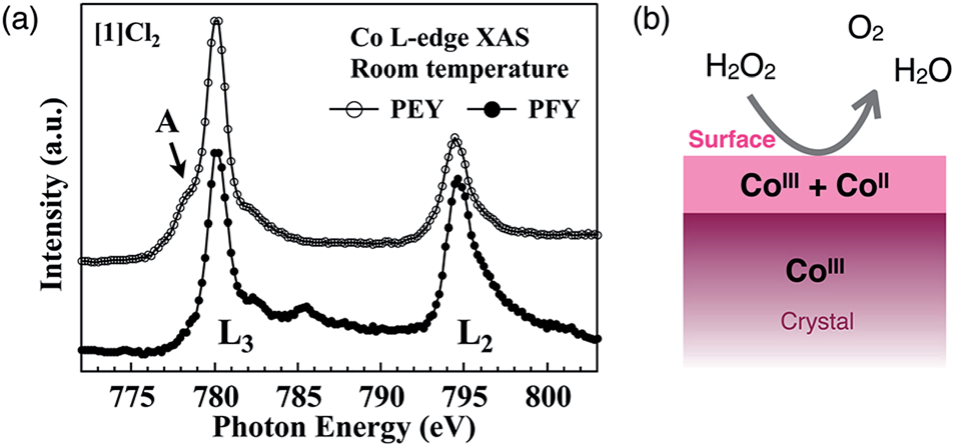
(a) Co L-edge XAS of [**1**]Cl_2_ measured in the PEY and PFY modes at 298 K and (b) the estimated oxidation states of Co in the bulk and at the surface of the crystal [**1**]X_*n*_.

Here, we reconsider the oxidation state of the cobalt centers by focusing on the crystal surface. Cobalt centers in [**1**]X_*n*_ have a +III oxidation state in the crystal as well as in solution. Consistent with this, in the partial fluorescence yield (PFY) mode of X-ray absorption spectroscopy (XAS), which is appropriate for bulk-layer observations, the crystal [**1**]Cl_2_ exhibited a single L_3_-edge peak at 780 eV due to cobalt(iii) species (Fig. 6a). However, in the partial electron yield (PEY) mode of XAS, which is appropriate for surface-layer observations, a clear shoulder (mark A) was observed at a lower energy of the L_3_-edge peak. The presence of this shoulder is indicative of the presence of cobalt(ii) species;^20–22^ therefore, the cobalt species located on the crystal surface at least partially exist in a +II oxidation state, although cobalt(iii) species are dominant in the bulk sample (Fig. 6b).^23^ This is supported by the EPR spectra of crystal [**1**]Cl_2_ at 4 K, which showed a very weak, broad signal at approximately *g* = 6 that is assigned to cobalt(ii).^24^ For [**2**]ClO_4_, which does not exhibit catalase-like activity, the EPR-silent state suggests the absence of cobalt(ii). It is quite reasonable that the aggregated cluster structure of inorganic anions in [**1**]X_*n*_ cannot be maintained on the crystal surface due to high repulsive energy among inorganic anions. This disruption of the aggregated anionic cluster structure should generate defects of inorganic anions, which in turn, leads to the reduction of cobalt(iii) to cobalt(ii) on the crystal surface to balance the total charge of the ionic crystal. Thus, the presence of cobalt species with a +II oxidation state, which are efficiently exposed at the (111) surface of the crystal, is key to the high catalase-like activity of the octahedral crystals [**1**]Cl_2_ and [**1**](NO_3_)_2_.

### The mechanism of the catalase-like reaction of [**1**]X_*n*_

**Scheme 1:**
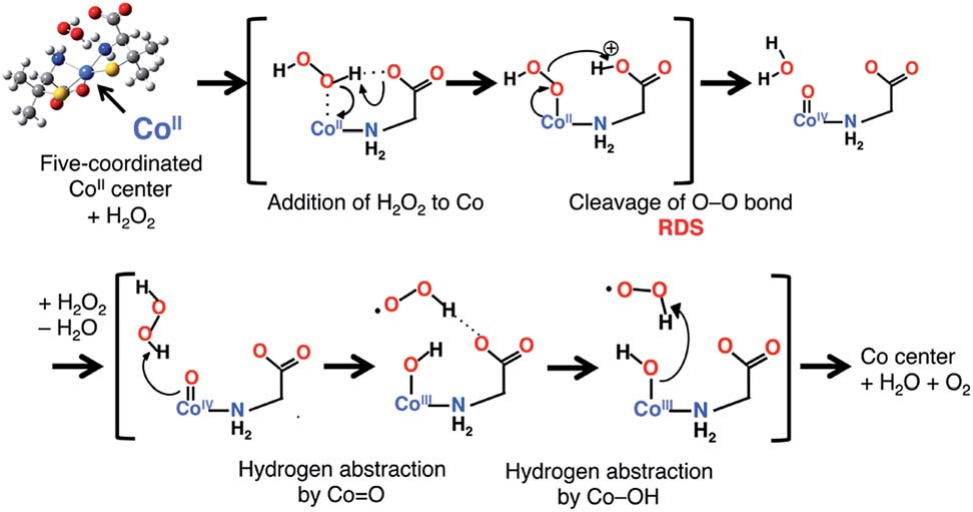
The proposed mechanism of the catalase-like catalytic reaction of [**1**]X_*n*_. Only the main parts of the Co center and substrates are shown for clarity.

The mechanism of the catalase-like reaction in this system was estimated as follows, based on the mechanism proposed for the iron(iii) center of native catalase^25^ but replacing the redox active site from iron to cobalt and the deprotonation site from histidine to carboxylate (Scheme 1). In the first step of this reaction, an H_2_O_2_ molecule approaches an octahedral cobalt(ii) center, with the concomitant dissociation of a carboxyl group to form a five-coordinated cobalt(ii) center. The carboxylate group acts as a base in the deprotonation of the H_2_O_2_ molecule, giving rise to a Co–OOH intermediate. The next step involves the formation of an oxo-cobalt(iv) center and the release of an H_2_O molecule *via* heterolytic O–O bond cleavage. Finally, another H_2_O_2_ molecule attacks the oxo-cobalt(iv) center to produce the original octahedral cobalt(ii) center, with the concomitant release of H_2_O and O_2_ molecules during the formation of a hydroxo-cobalt(iii) center and an HOO˙ radical. Each transition state was calculated with the effect of solvent (H_2_O) at the UB3LYP-D/6-31G* level,^26^ which revealed that the O–O bond cleavage is the rate-determining step (RDS) with an activation energy of 21.2 kcal mol^–1^ relative to the substrate complex (Fig. S14†). We also calculated each transition state of the cobalt(iii) model, in which the cobalt(ii) center is replaced by a cobalt(iii) center. In the cobalt(iii) model, the activation energy of the RDS (O–O bond cleavage) was calculated as 26.7 kcal mol^–1^, which is higher than that in the cobalt(ii) model (Fig. S15†). This result indicates that the cobalt(ii) model is preferable for this catalase-like reaction.

## Conclusions

We showed that [**1**]X_*n*_ ionic crystals, which consist of Au^I^_4_Co^III^_2_ complex cations ([**1**]^2+^) and inorganic anions (X = Cl^–^, NO_3_^–^, ClO_4_^–^, and SO_4_^2–^), act as heterogeneous catalysts for a catalase-like reaction. To our knowledge, this is the first example of cobalt(iii) coordination compounds that exhibit catalase-like activity under heterogeneous conditions. Of note is the remarkably high catalytic activity, which is dependent on the crystal shape (octahedral *vs.* cubic) rather than the charge (–1 *vs.* –2) and the geometry of the inorganic anions. This is due to the unusual arrangement of cations and anions in the crystal [**1**]X_*n*_, leading to the generation of cobalt(ii) centers on the crystal surface, which are efficiently exposed on the (111) plane with readily dissociated carboxyl groups compared to the (100) plane. This study demonstrated that the oxidation state and exposure of metal centers on the crystal surface that are required for heterogeneous catalytic activity can be controlled *via* the arrangement of cationic and anionic components as well as the crystal shape and morphology. These results provide significant insight into the future design and creation of various heterogeneous catalysts based on ionic crystals.

## Experimental

### Materials

Chemicals were purchased from commercial sources and used without further purification. Aqueous solutions of hydrogen peroxide (30 wt%) were purchased from Wako Pure Chemical Industries. Ultra-pure water was obtained from a Millipore Direct-Q3 UV water purification system, wherein the electronic conductance was 18.2 MΩ cm^–1^. [Au^I^_4_Co^III^_2_(dppe)_2_(d-pen)_4_]X_*n*_ ([**1**]X_*n*_, X_*n*_ = (Cl^–^)_2_, (ClO_4_^–^)_2_, (NO_3_^–^)_2_ or SO_4_^2–^), [Au_4_Cr_2_(dppe)_2_(d-pen)_4_]Cl_2_, [Co^III^{Au^I^(PPh_3_)(d-pen)}_2_]ClO_4_ ([**2**]ClO_4_) and [Au_2_(dppe)(d-Hpen)_2_] were prepared according to the methods reported in the literature.^11a,18^

### Catalase-like catalytic disproportionation of hydrogen peroxide

A typical procedure for the catalase-like catalytic disproportionation of hydrogen peroxide is as follows. A vial containing 1.0 mL of 5 wt% aqueous H_2_O_2_ and another vial containing [**1**]Cl_2_ (5.0 mg) were sealed with rubber septa. The two vials were carefully deaerated by bubbling Ar for 15 min. The aqueous H_2_O_2_ solution was transferred to the vial containing crystals *via* a Teflon tube to start the reaction without stirring at 298 K. At each reaction time, 100 μL of Ar gas was injected into the vial, and the same volume of gas in the headspace was then sampled by a gastight syringe and quantified on a Shimadzu GC-17A gas chromatograph (GC) [Ar carrier gas, capillary column with molecular sieves (Agilent Technologies 19094PMS0, 30 m × 0.53 mm), 313 K] equipped with a thermal conductivity detector (TCD). Microscopic observation of the crystal sizes and filtration treatment were performed as needed.

### ICP-AES measurements

ICP-AES was measured on a Shimadzu ICPS-8100. The samples were obtained by filtration of the reaction mixture at 20–30 min in conditions similar to the reaction for GC measurements.

### XPS measurements

XPS spectra were measured on a Kratos Axis 165x with a 165 mm hemispherical electron energy analyzer. The incident radiation was a Mg-Kα X-ray (1253.6 eV) at 120 W (12 kV, 10 mA), and a charge neutralizer was turned on for acquisition. Each sample was mounted on a stainless-steel stage with double-sided carbon scotch tape. The binding energy of each element was corrected using the C 1s peak (284.5 eV) from the residual carbon.

### NMR measurements

^1^H and ^31^P NMR spectra were measured on an ECA 500 spectrometer at 298 K. The NMR spectra were calibrated with TMS (methanol-*d*_4_) = 0 ppm as an internal standard.

### PXRD measurements

High-quality powder X-ray diffractions were recorded at room temperature, in transmission mode [synchrotron radiation *λ* = 0.999139(2) Å; 0° ≤ 2*θ* ≤ 30°; step width = 0.006°; data collection time = 30 s] on a diffractometer equipped with a MYTHEN microstrip X-ray detector (Dectris Ltd.) at the SPring-8 BL02B2 beamline. The crystals were loaded into glass capillary tubes (diameter = 0.3 mm), and the samples were rotated during the measurements.

### SEM measurements

SEM observations were performed on an FEI Magellan 400L instrument at 5 × 10^–5^ Pa. The working distance was set to 1.9–2.7 mm. Secondary electrons were collected with an Everhart-Thornley detector. The observations were performed at a beam landing voltage of 200 or 1000 V, and 200 V imaging was performed by applying 800 V of the beam deceleration bias onto the sample against the primary electron beam accelerated at 1 kV.

### EPR measurements

The sample for EPR was prepared by flowing N_2_ for 15 min through a Teflon tube to the crystals in a quartz EPR tube (2.0 mm i.d.). EPR spectra of the solutions were obtained on a JEOL X-band spectrometer (JES-FA200) at 4 K. The *g* value was calibrated using a Mn^2+^ marker. The EPR spectra were obtained under non-saturating microwave power conditions. The magnitude of modulation was chosen to optimize the resolution and signal-to-noise (S/N) ratio of the observed spectra.

### XAS measurements^21^

The XAS measurements were carried out at BL-11 of the Synchrotron Radiation Center at Ritsumeikan University, Japan. In this beamline, so-called varied-line-spacing plane gratings were employed to supply monochromatic photons with *hν* = 40–1000 eV. The Co L_2,3_-edge XAS spectra (*hν* = 760–810 eV), were taken simultaneously in the partial fluorescence yield (PFY) and partial electron yield (PEY) modes with a photon energy resolution of ∼300 meV. For the Co L_2,3_-edge XAS measurements, the luminescence with *hν* = 700–950 eV, including the Co L lines, was detected as a signal. On the other hand, in PEY mode, the microchannel plate (MCP) for detecting the Auger and secondary electrons was set in the 45°-depression to the photon propagation. On the front of the MCP, a gold mesh was installed to enable the application of a voltage. We applied the voltage of –550 V to the mesh for the Co L_2,3_-edge measurements in order to suppress a strong background caused by the C, N and O K-edge absorptions in the spectra. The powder-like single-crystalline samples of [**1**]Cl_2_ and [**2**]ClO_4_ were thinly expanded on the conductive carbon tape attached on the sample holder in air before transferring them to the vacuum chamber. We repeatedly measured the spectra on the same and different sample positions to confirm the data reproducibility with neither serious radiation damage nor a sample-position dependence of the Co L_2,3_-edge XAS spectra. The measurements were performed at room temperature. Energy values were calibrated with the top of the L_3_-edge peak of LiCo^III^O_3_ (780.32 eV).

### Theoretical calculations

Geometry optimizations were carried out at the UB3LYP-D/6-31G* level of theory for each transition state, as implemented in Gaussian 09, revision C.01.^26*a*^ The effect of solvent (H_2_O) was included implicitly using the polarizable continuum model (PCM)^26*c*^ with the cavity constructed by the universal force-field (UFF) bond radii. The charges and spin multiplicities of the Co center models in the initial state were –2 and 4 for the Co^II^ center model and –1 and 1 for the Co^III^ center model, respectively.
