# Supplementary material for: Heterogeneous catalase-like activity of gold(i)–cobalt(iii) metallosupramolecular ionic crystals
Source: Chem Sci. 2017 Jan 16;8(4):2671–6. doi: 10.1039/c6sc04993a (PMC5433492; doi:10.1039/c6sc04993a)
Supplement: Supplementary file 1 [file SC-008-C6SC04993A-s001.pdf]

Electronic Supplementary Material (ESI) for Chemical Science.
This journal is © The Royal Society of Chemistry 2017

Electronic Supplementary Information

Heterogeneous Catalase-like Activity of Gold(I)-Cobalt(III) Metallosupramolecular Ionic Crystals

M. Yamada,[a] N. Yoshinari,[a] N. Kuwamura,[a] T. Saito,[b] S. Okada,[c] S. P. Maddala,[c] K. Harano,[c] E. Nakamura,[c] K. Yamagami,[d] K. Yamanaka,[e] A. Sekiyama,[d] T. Suenobu,[f] Y. Yamada,[g] and T. Konno*[a]

[a]Department of Chemistry, Graduate School of Science, Osaka University, Toyonaka, Osaka 560-0043 (Japan)

Fax: (+81) 6-6850-5765

E-mail: konno@chem.sci.osaka-u.ac.jp

[b]Department of Biomedical Information Science, Graduate School of Information Science, Hiroshima City University, Asa-Minami-ku, Hiroshima 731-3194 (Japan)

[c]Department of Chemistry, Graduate School of Science, The University of Tokyo, Bunkyo-ku, Tokyo, 113-0033 (Japan)

[d]Division of Materials Physics, Graduate School of Engineering Science, Osaka University, Toyonaka, Osaka 560-8531 (Japan)

[e]Synchrotron Radiation Center, Ritsumeikan University, Kusatsu, Shiga 525-8577 (Japan)

[f]Department of Material and Life Science, Graduate School of Engineering, Osaka University, ALCA and SENTAN, Japan Science and Technology (JST), Suita, Osaka 565-0871 (Japan)

[g]Department of Applied Chemistry & Bioengineering, Graduate School of Engineering, Osaka City University, 3-3-138 Sugimoto, Sumiyoshi-ku, Osaka 558-8585 (Japan)

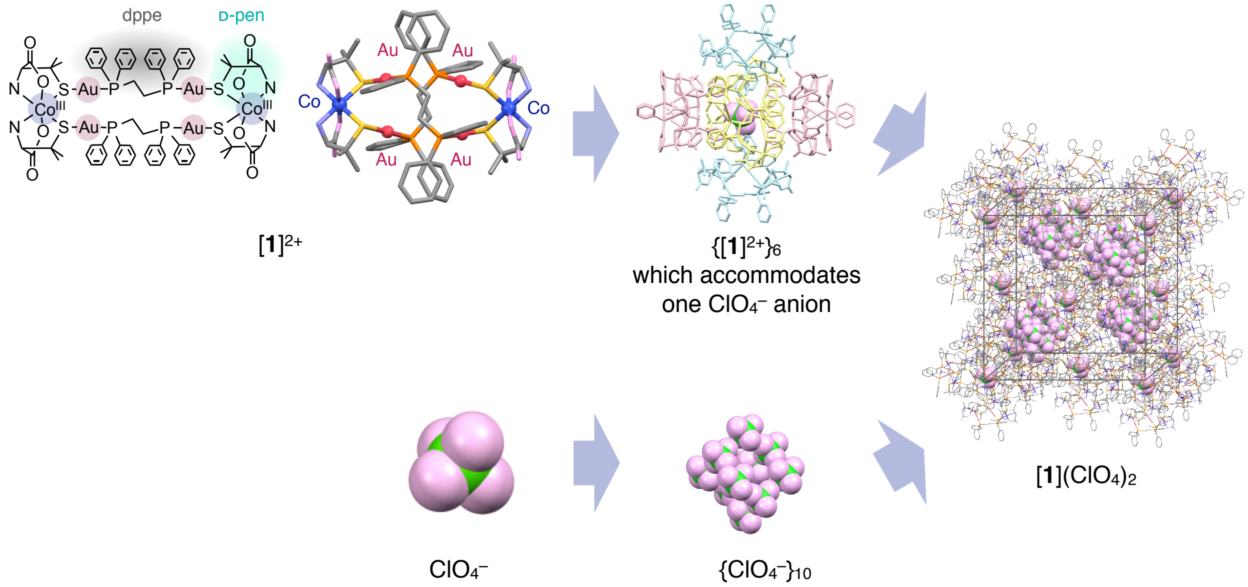

Fig. S1 Schematic figure of the formation of [**1**]$(ClO_4)_2$. H_2O molecules are omitted for clarity.

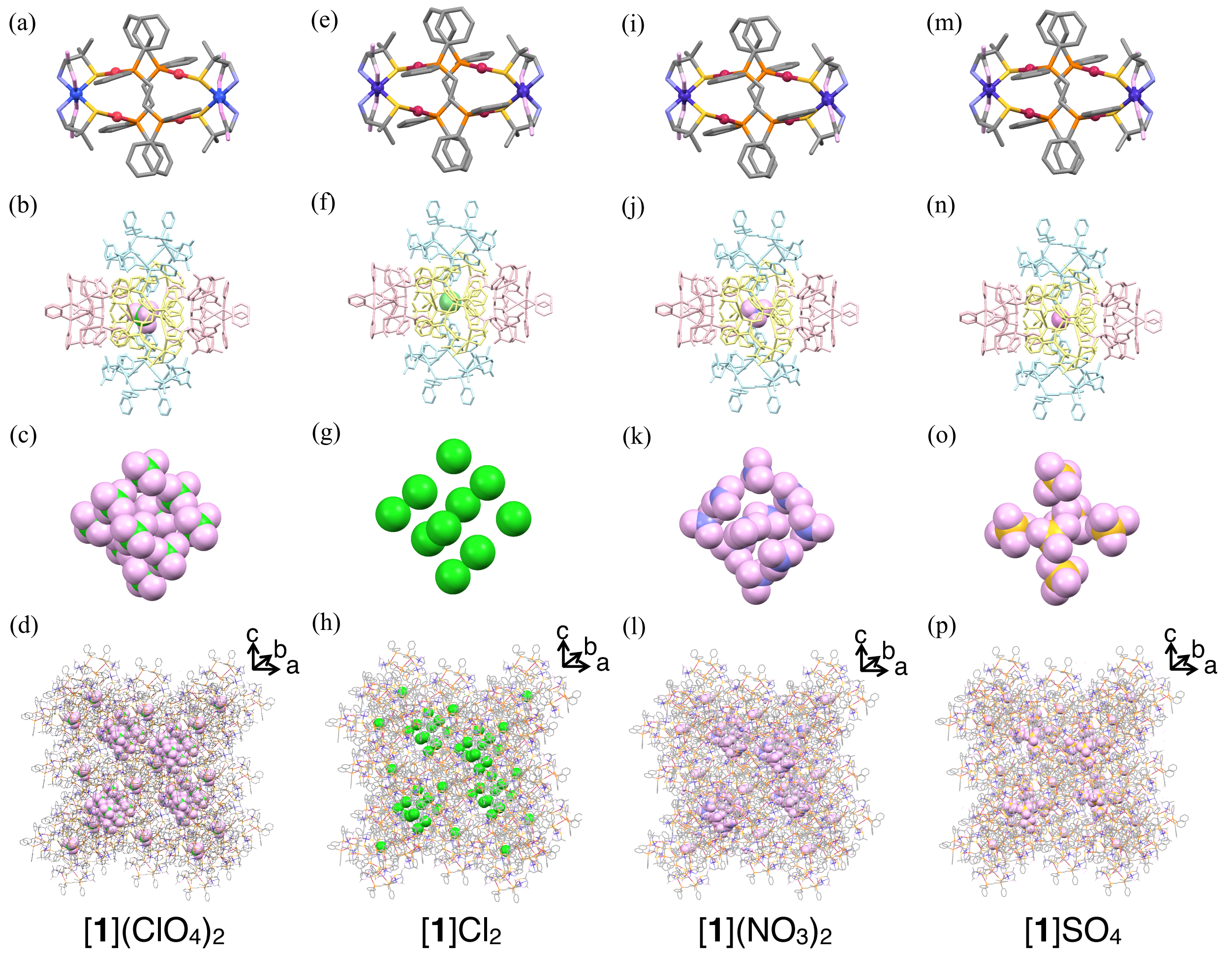

Fig. S2 Structures of (a, e, i, m) complex cations, (b, f, j, n) octahedron-shaped cationic supramolecules $\{[\mathbf{1}]^{2+}\}_6$ accommodating an anion, (c, g, k) adamantane-shaped anionic clusters $\{X^{-}\}_{10}$ and (o) octahedron-shaped anionic clusters $\{X^{2-}\}_6$ and (d, h, l, p) the packing structure of $[\mathbf{1}](ClO_4)_2$, $[\mathbf{1}]Cl_2$, $[\mathbf{1}](NO_3)_2$, and $[\mathbf{1}]SO_4$, respectively.

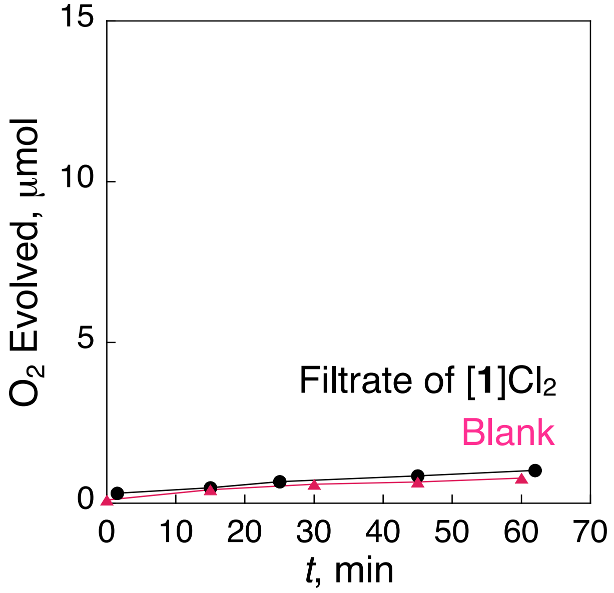

Fig. S3 Time profiles of the evolution of O_2 from the filtrate of [**1**]Cl_2 (1 mL) (black circles) and 5 wt% aqueous H_2O_2 (1 mL) (pink triangles) at 298 K. The filtrate of [**1**]Cl_2 was prepared by the following procedure; the catalytic amount of crystals of [**1**]Cl_2 (4.7 mg, approximately 1.0 × 1.0 × 1.0 mm) was soaked in a 5 wt% aqueous H_2O_2 (1 mL) and filtered after 20 min.

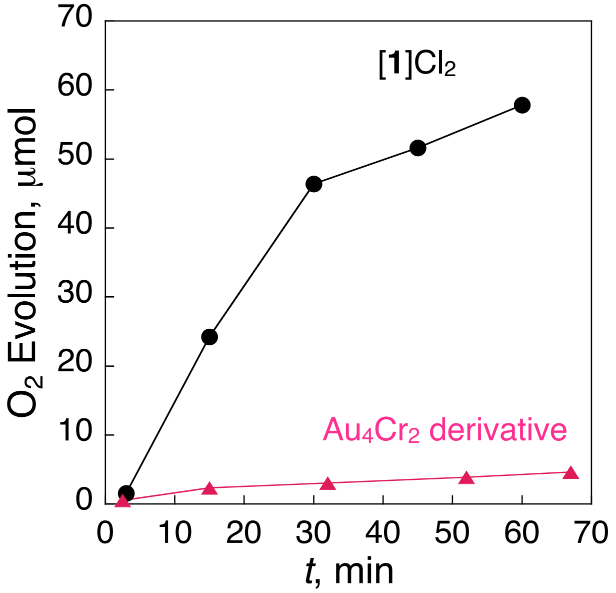

Fig. S4 Time profiles of the evolution of O_2 during treatment with a catalytic amount of [**1**]Cl_2 (5.0 mg, approximately 0.5 × 0.5 × 0.5 mm) and the Au_4Cr_2 derivative [Au_4Cr_2(dppe)$_2$(D-pen)$_4$]Cl_2 (5.2 mg, approximately 0.05 × 0.05 × 0.05 mm) with 5 wt% aqueous H_2O_2 (1.00 mL) at 298 K.

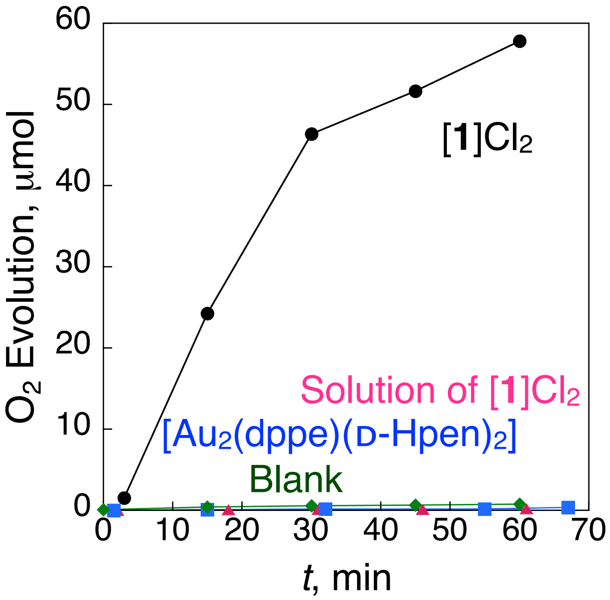

Fig. S5 Time profiles of the evolution of O_2 in the disproportionation of 5 wt% aqueous H_2O_2 with a catalytic amount of crystals of [**1**]Cl_2 (5.0 mg, approximately 0.5 × 0.5 × 0.5 mm), a saturated homogeneous solution of [**1**]Cl_2 and a metalloligand [Au_2(dppe)(D-Hpen)$_2$] (5.2 mg) and without the addition of any metal complex in H_2O (1.00 mL) at 298 K.

(a)

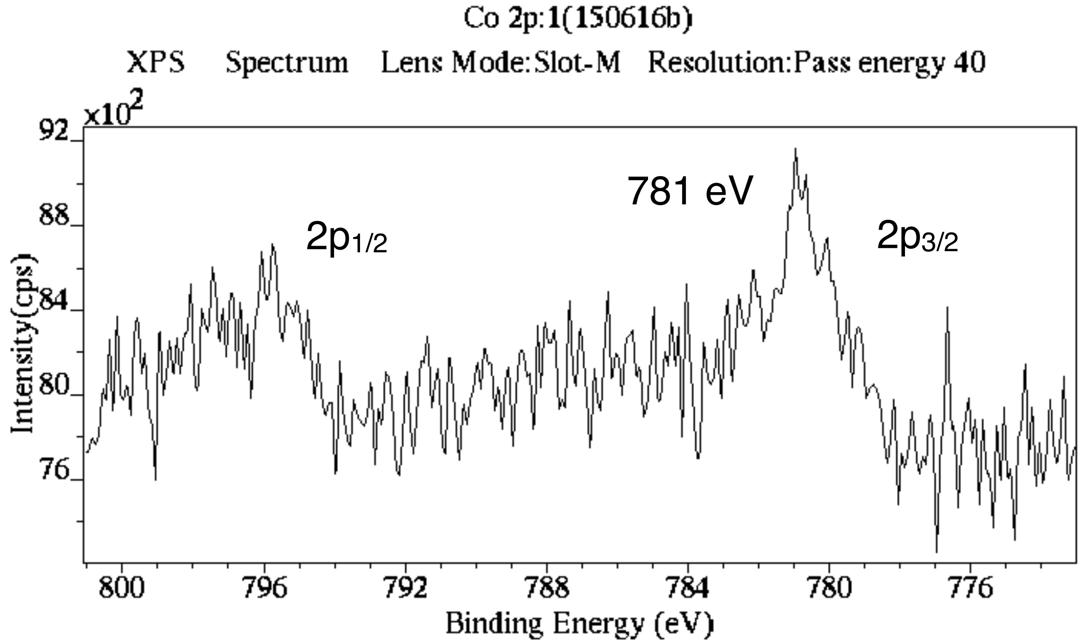

(b)

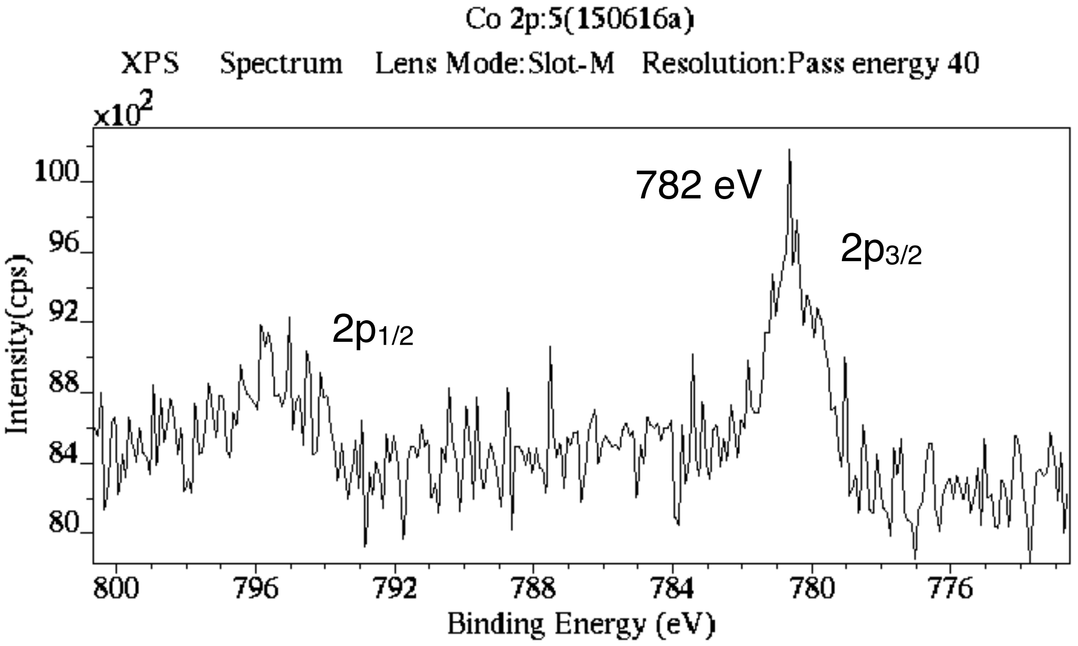

Fig. S6 XPS of [**1**]Cl_2 (a) before and (b) after reaction with H_2O_2 for 15 min.

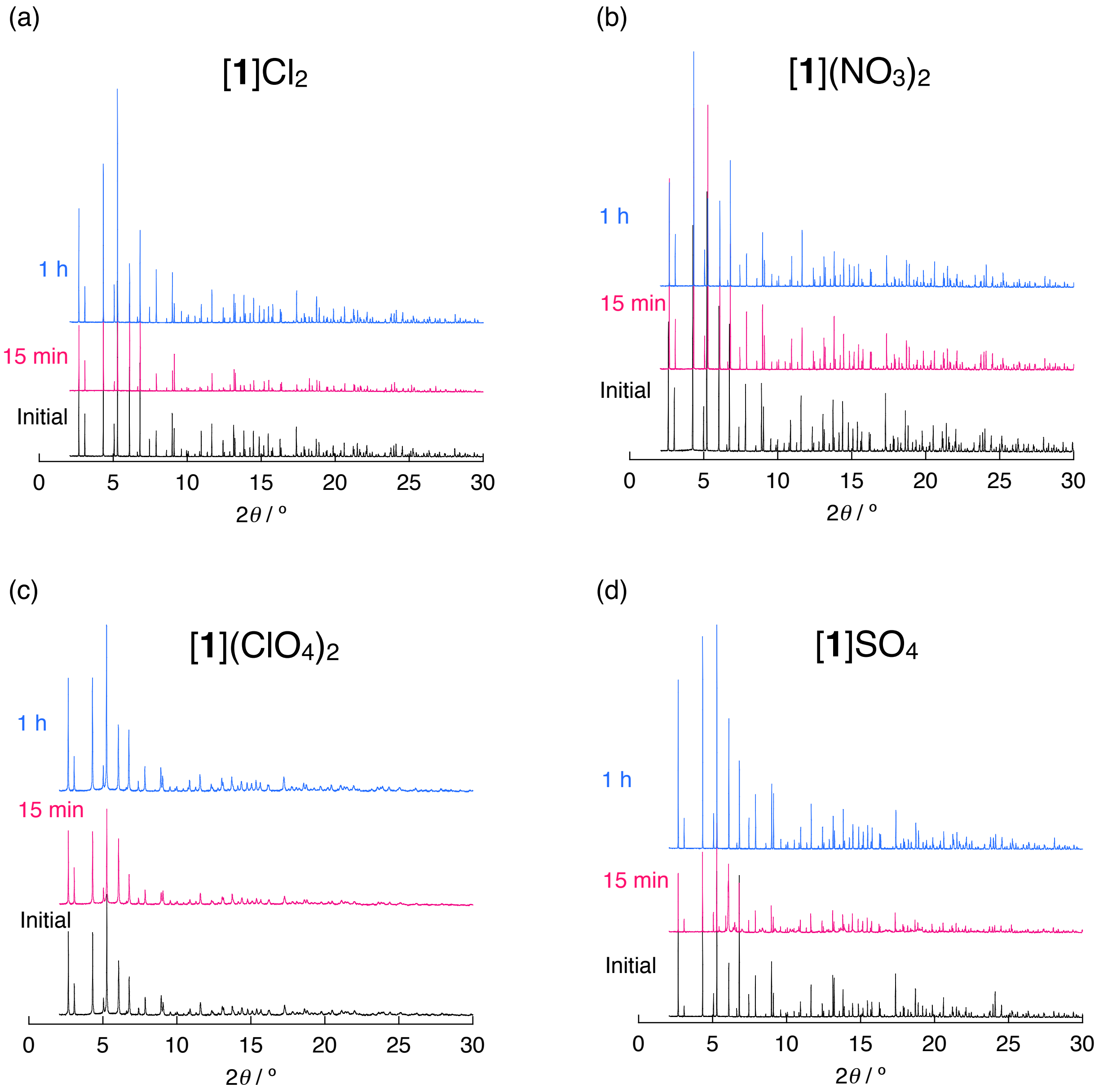

Fig. S7 Powder XRD patterns of the initial samples and samples reacted with H_2O_2 for 15 min or 1 h for (a) [**1**]Cl_2, (b) [**1**]$(NO_3)_2$, (c) [**1**]$(ClO_4)_2$ and (d) [**1**]SO_4.

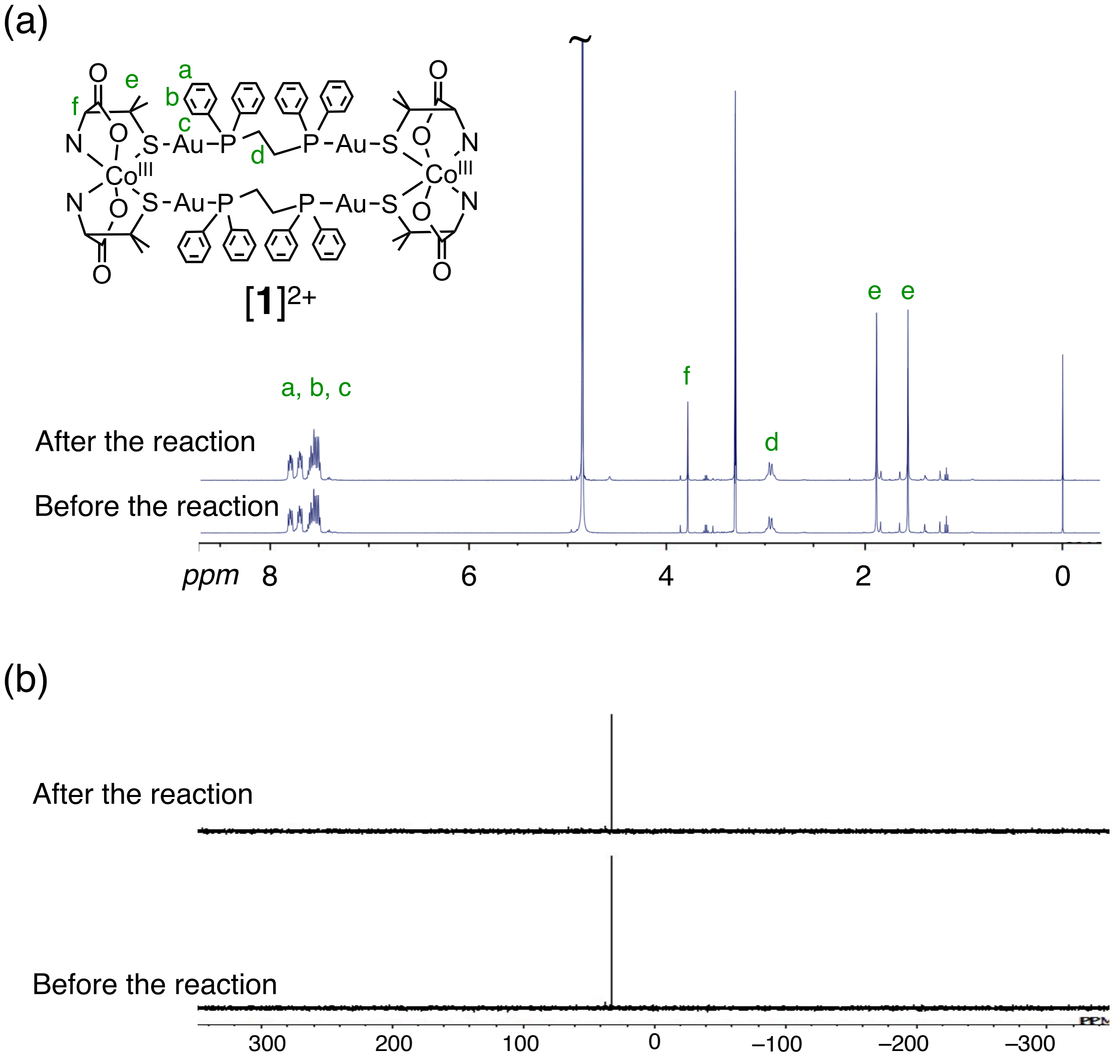

Fig. S8 (a) ^{1}H and (b) ^{31}P NMR spectra (500 MHz for ^{1}H and 202 MHz for ^{31}P, methanol-d_4, 298 K) of [**1**]Cl_2 before and after reaction with H_2O_2 for 15 min.

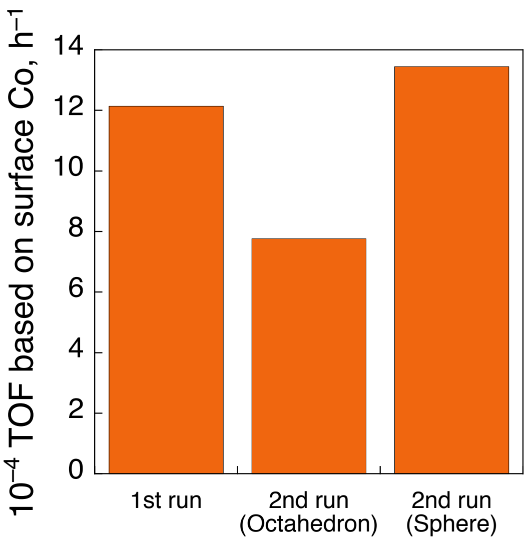

Fig. S9 TOF (h^{-1}) calculated based on the surface Co atoms at 15 min in the disproportionation of 5 wt% aqueous H_2O_2 with [**1**]Cl_2 (1st: 5.2 mg, 1.0 × 1.0 × 1.0 mm; 2nd: 4.6 mg, 0.3 × 0.3 × 0.3 mm) in H_2O (1.00 mL) at 298 K in the first run and second run. In the second run, the calculation was carried out with an approximation of the complete octahedral crystal in the initial state and the complete spherical crystal with the lowest specific surface area to show a possible range because the crystals obtained after the first run were not complete octahedrons due to partial cracking.

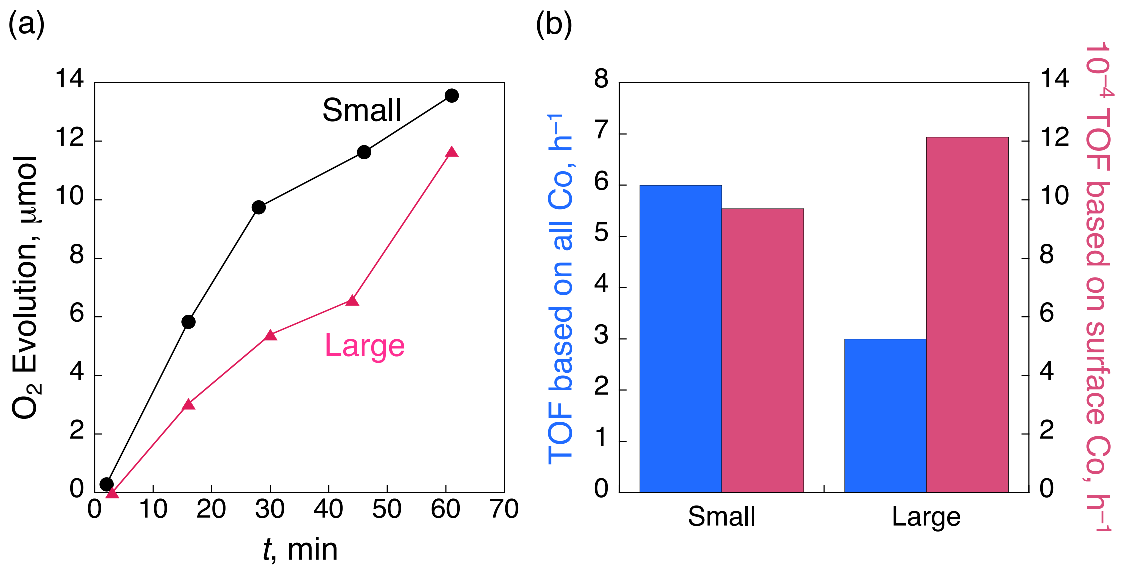

Fig. S10 (a) Time profiles of the evolution of O_2 in the disproportionation of 5 wt% aqueous H_2O_2 with a catalytic amount of small crystals (5.2 mg, 0.4 × 0.4 × 0.4 mm) and large crystals (5.2 mg, 1.0 × 1.0 × 1.0 mm) of [**1**]Cl_2 in H_2O (1.00 mL) at 298 K and (b) the TOF calculated based on all the Co atoms or the surface Co atoms at 15 min.

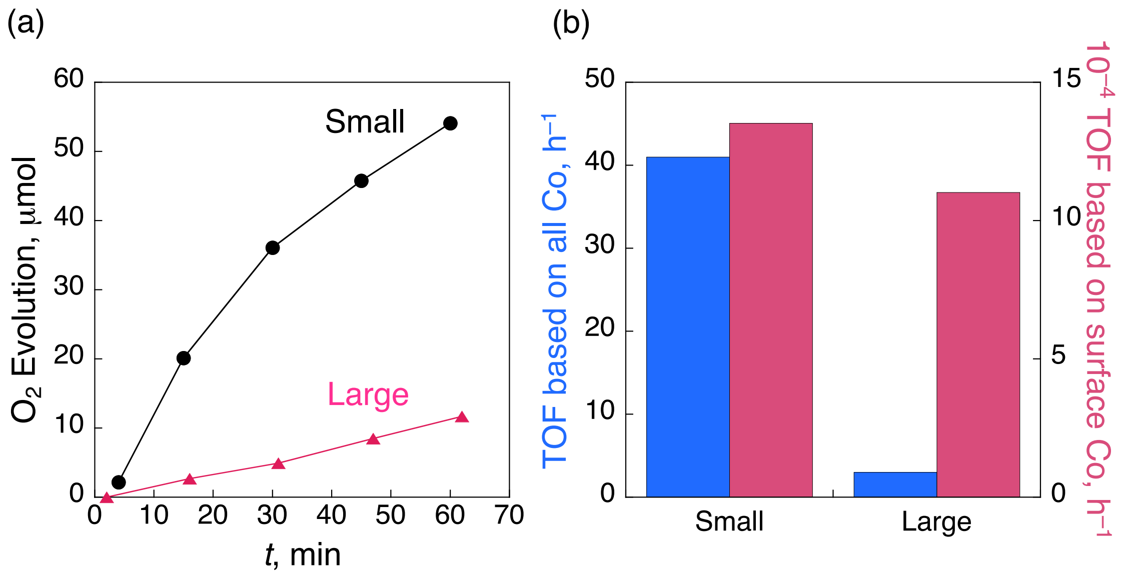

Fig. S11 (a) Time profiles of the evolution of O_2 in the disproportionation of 5 wt% aqueous H_2O_2 with a catalytic amount of small crystals (4.9 mg, 0.05 × 0.05 × 0.05 mm) and large crystals (5.1 mg, 1.0 × 1.0 × 1.0 mm) of [**1**]$(NO_3)_2$ in H_2O (1.00 mL) at 298 K and (b) the TOF calculated based on all the Co atoms or the surface Co atoms at 15 min.

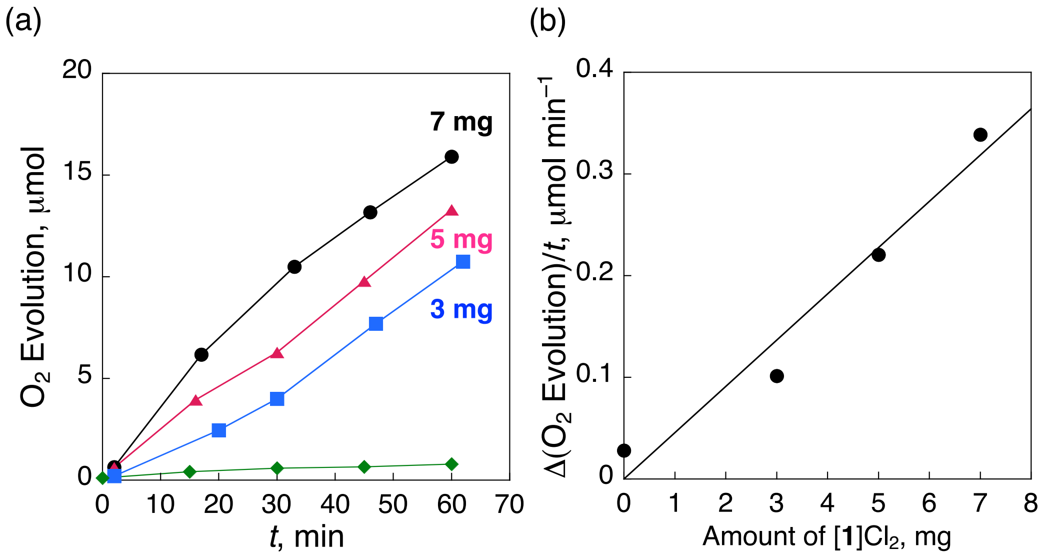

Fig. S12 (a) Time profiles of the evolution of O_2 in the disproportionation of 5 wt% aqueous H_2O_2 with various amounts of 1.0 × 1.0 × 1.0 mm-sized crystals of [**1**]Cl_2 (3.0, 4.8, 7.6 and 0 mg) in H_2O (1.00 mL) at 298 K and (b) the dependence on the amount of [**1**]Cl_2 of the initial rate of evolution of O_2, Δ(O_2 evolution)/*t*, calculated at 15 min .

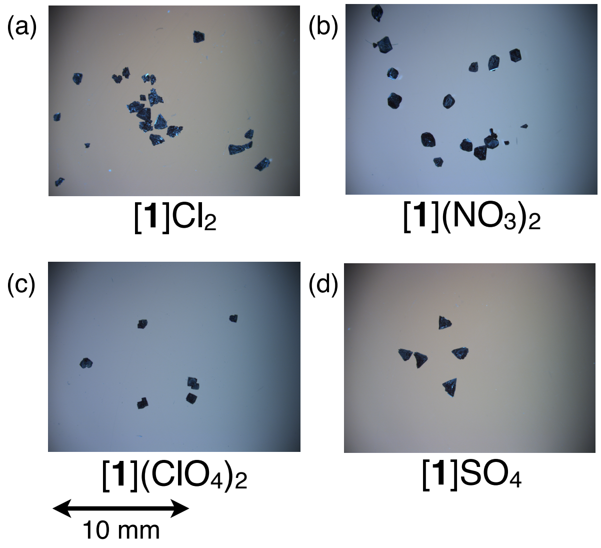

Fig. S13 Microscopic photos of crystals of (a) [**1**]Cl_2, (b) [**1**]$(NO_3)_2$, (c) [**1**]$(ClO_4)_2$ and (d) [**1**]SO_4 used for the reaction.

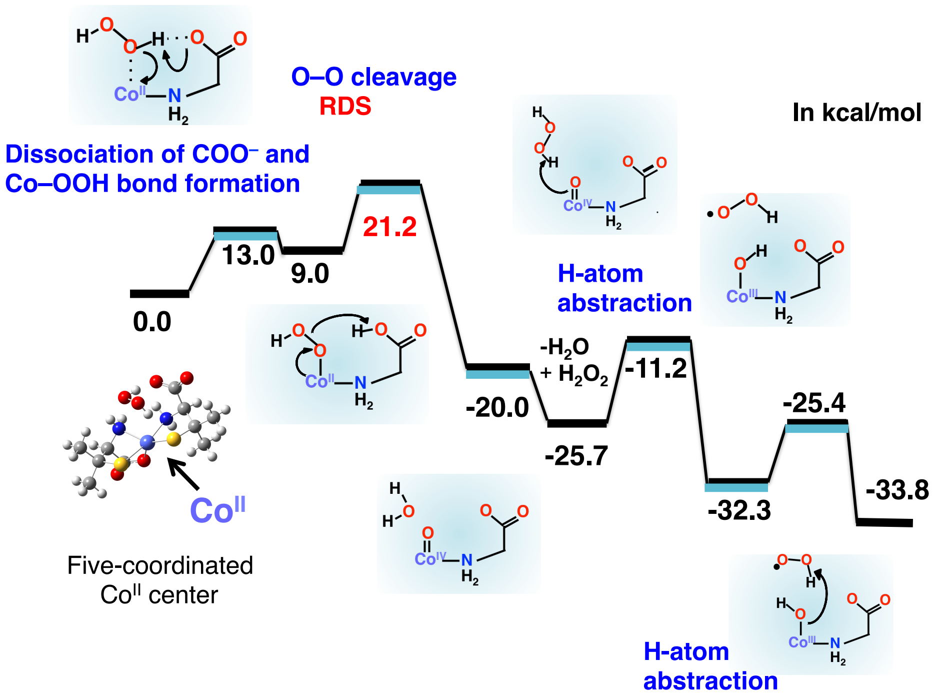

Fig. S14 Energy profile of the proposed mechanism from a five-coordinated Co^{II} center. Optimization of the models and calculation of the relative energies (in kcal/mol) were performed at the UB3LYP-D/6-31G* level.

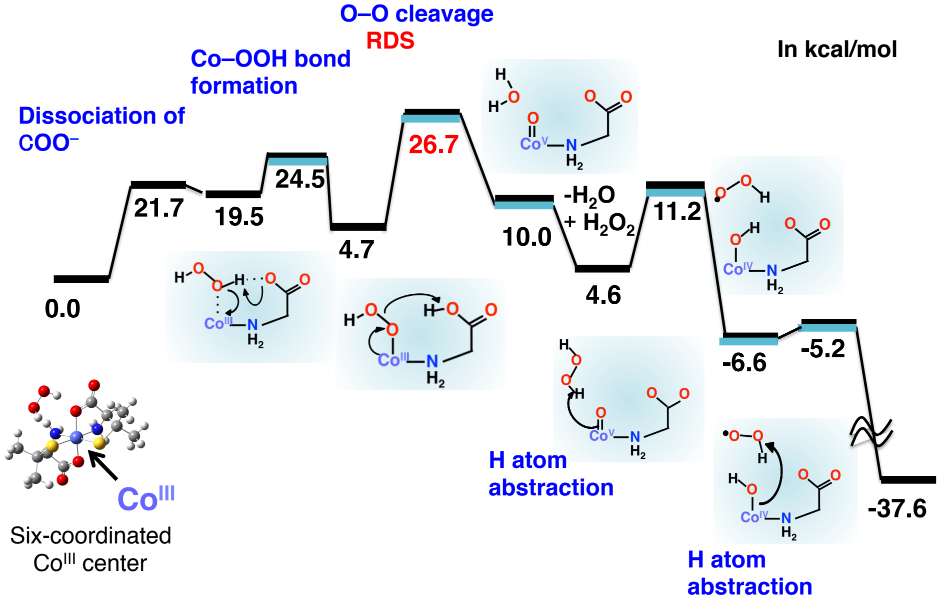

Fig. S15 Energy profile of the proposed mechanism starting from a six-coordinated Co^{III} center. Optimization of the models and calculation of the relative energies (in kcal/mol) were performed at the UB3LYP-D/6-31G* level.

Table S1 Concentrations of saturated aqueous solutions of [**1**]X_n estimated from UV-vis spectra based on the molar absorption coefficient of [**1**]Cl_2 in a methanol solution.

Compound	Solubility
$[Au^{I}_4Co^{III}_2(dppe)_2(\text{D-pen})_4]Cl_2$	0.18 mM
$[Au^{I}_4Co^{III}_2(dppe)_2(\text{D-pen})_4](NO_3)_2$	< 0.01 mM
$[Au^{I}_4Co^{III}_2(dppe)_2(\text{D-pen})_4](ClO_4)_2$	0.11 mM
$[Au^{I}_4Co^{III}_2(dppe)_2(\text{D-pen})_4]SO_4$	1.4 mM

Table S2 Concentrations of leached Co and Au in the reaction mixture at 20-30 min detected by ICP-AES after filtration of the crystals [1]X_n. The reaction conditions are similar to the reactions for GC measurements: 5 wt% aqueous H_2O_2 with a catalytic amount of crystals of [**1**]Cl_2 (75.0 mg, approximately 1.0 × 1.0 × 1.0 mm) or [**1**]$(NO_3)_2$ (74.9 mg, approximately 1.0 × 1.0 × 1.0 mm) in H_2O (15.0 mL) at 298 K.

Compound	Concentration of Co (Leached crystal ratio)	Concentration of Au (Leached crystal ratio)
$[Au^{I}_4Co^{III}_2(dppe)_2(\text{D-pen})_4]Cl_2$	0.435 ppm (0.188%)	1.99 ppm (0.128%)
$[Au^{I}_4Co^{III}_2(dppe)_2(\text{D-pen})_4](NO_3)_2$	0.052 ppm (0.023%)	0.25 ppm (0.016%)

Table S3 Representatives of the reported catalase-like activities.

Catalyst	Catalytic activity[d]	Conditions
Typical heme catalases[a]	TOF = $5.4 \times 10^4 \sim 83.3 \times 10^4$ s^{-1}	various
MnO_2[b]	$k = 2.3 \times 10^4$ s^{-1}	rt, [H_2O_2] = 0.50 M, MnO_2 (50 mg) in 250 mL reactor, at 0-100 min
$K_2Cr_2O_7$[b]	$k = 1.2 \times 10^4$ s^{-1}	rt, [H_2O_2] = 0.50 M, $K_2Cr_2O_7$ (50 mg) in 250 mL reactor, at 0-100 min
Co(HPHOPD)$_2$[c]	TOF = 5.5×10^3 h^{-1}	308 K, [H_2O_2] = 5 vol, Co(HPHOPD)$_2$ (40 mg) in 10 mL, at 10 min
[**1**]Cl_2	TOF = 1.2×10^5 h^{-1} (= ~3.3×10 s^{-1}) k = 8.0 s^{-1}	rt, [H_2O_2] = 5%, [**1**]Cl_2 (5 mg) in 1 mL, at 15 min
[**1**](NO_3)$_2$	TOF = 1.4×10^5 h^{-1} (= ~3.9×10 s^{-1}) k = 8.2 s^{-1}	rt, [H_2O_2] = 5%, [**1**](NO_3)$_2$ (5 mg) in 1 mL, at 15 min

[a]J. Switala and P. C. Loewen, *Arch. Biochem. Biophys.*, 2002, **401**, 145–154; J. Vlasits, C. Jakopitsch, M. Bernroitner, M. Zamocky, P. G. Furtmüller and C. Obinger, *Arch. Biochem. Biophys.*, 2010, **500**, 74–81. [b]M.A. Hasnat, M. M. Rahman, S.M. Borhanuddin, A. Siddiqua, N.M. Bahadur and M.R. Karim, *Catal. Comm.*, 2010, **12**, 286–291. [c]HOHOPD = 2-oximinopropanedione, R. V. Prasad and N. V. Thakkar, *J. Mol. Catal.*, 1994, **92**, 9–20. [d]The values of TOF and k were calculated as V/[catalyst] and $2V$/[H_2O_2], where V = O_2 evolution rate.
